# Comparative Studies on the Photoreactivity, Efficacy, and Safety of Depigmenting Agents

**DOI:** 10.3390/ph17010055

**Published:** 2023-12-28

**Authors:** Sandra Mota, Gonçalo P. Rosa, Maria Carmo Barreto, Jorge Garrido, Emília Sousa, Maria T. Cruz, Isabel F. Almeida, Clara Quintas

**Affiliations:** 1 UCIBIO—Applied Molecular Biosciences Unit, MedTech, Faculty of Pharmacy, Department of Drug Sciences, Laboratory of Pharmaceutical Technology, University of Porto, 4050-313 Porto, Portugal; up201608486@up.pt; 2 Associate Laboratory i4HB—Institute for Health and Bioeconomy, Faculty of Pharmacy, University of Porto, 4050-313 Porto, Portugal; claraquintas@ff.up.pt; 3 LAQV-REQUIMTE, Department of Chemistry, University of Aveiro, 3810-192 Aveiro, Portugal; goncalo.p.rosa@uac.pt; 4 cE3c—Centre for Ecology, Evolution and Environmental Changes/Azorean Biodiversity Group, CHANGE and Faculty of Sciences and Technology, University of Azores, 9500-321 Ponta Delgada, Portugal; 5 CIQUP-IMS, ISEP, Polytechnic of Porto, 4249-015 Porto, Portugal; jjg@isep.ipp.pt; 6 Laboratory of Organic and Pharmaceutical Chemistry, Department of Chemical Sciences, Faculty of Pharmacy, University of Porto, 4050-313 Porto, Portugal; esousa@ff.up.pt; 7 CIIMAR—Interdisciplinary Center of Marine and Environmental Research, University of Porto, Avenida General Norton de Matos, S/N, 4450-208 Matosinhos, Portugal; 8 Faculty of Pharmacy, University of Coimbra, 3000-548 Coimbra, Portugal; trosete@ff.uc.pt; 9 CNC-UC—Center for Neuroscience and Cell Biology, University of Coimbra, 3000-548 Coimbra, Portugal; 10 CIBB—Centre for Innovative Biomedicine and Biotechnology, University of Coimbra, 3000-548 Coimbra, Portugal; 11 UCIBIO—Applied Molecular Biosciences Unit, Faculty of Pharmacy, Department of Drug Sciences, Laboratory of Pharmacology, University of Porto, 4050-313 Porto, Portugal

**Keywords:** depigmenting agents, cosmetic, skin pigmentation, photoreactivity, tyrosinase inhibition, melanocytes, cytotoxicity

## Abstract

Depigmenting products are increasingly used to counteract skin hyperpigmentation and related psychosocial issues. This study aimed to compare different depigmenting agents—4-butylresorcinol; bakuchiol; tranexamic acid; ascorbyl glucoside; α-arbutin; and ascorbic acid—for photoreactivity; tyrosinase inhibition; and safety. Photoreactivity was assessed using the Reactive Oxygen Species assay. In vitro tyrosinase inhibition was compared, and cell viability was assessed in B-16V melanocytes to evaluate safety. Results showed 4-butylresorcinol, ascorbyl glucoside, and α-arbutin are non-photoreactive, while for ascorbic acid and bakuchiol it was not possible to reach conclusive results due to the lack of specificity of the ROS assay. 4-Butylresorcinol, acting as a competitive inhibitor, displayed potent tyrosinase inhibition, followed by ascorbic acid and bakuchiol. Both 4-butylresorcinol and bakuchiol reduced cell viability in a concentration-dependent manner. The insights obtained in this work support the development of depigmenting products by providing useful scientific guidance on the photostability, tyrosinase inhibitory efficacy, and skin safety of depigmenting agents.

## 1. Introduction

Consumers are increasingly looking for depigmenting cosmetic solutions as skin hyperpigmentation disorders have a significant impact on quality of life [1,2]. Furthermore, the increase in life expectancy, along with a growing demand for solutions to combat the effects of aging on the skin, has given rise to a prevalent concern related to aging skin. Among the common symptoms associated with aging skin, abnormal pigmentation, including mottled pigmentation (senile lentigo) and melasma, stands out. In accordance, the overall market for skin-lightening cosmetic products is anticipated to reach near USD 17 billion by 2030, growing at a compound annual growth rate of 5.5% between 2022 and 2030 [3]. In Europe, the market for depigmenting cosmetic products is expected to grow at a compound annual growth rate of 4.8% between 2022 and 2028 [4].

**Figure 1:**
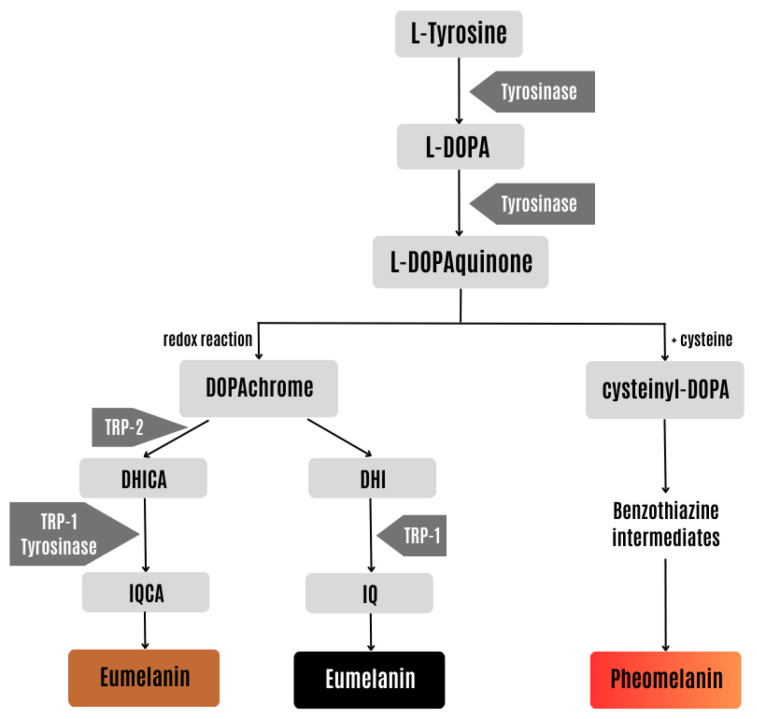
Synthesis of the two types of melanin: eumelanin (brown and black) and pheomelanin (yellow-red). L-DOPA, 3,4-dihydroxy-L-phenylalanine; TRP-1, tyrosinase-related protein 1; TRP-2, tyrosinase-related protein 2; DHICA, 5,6-dihydroxy indole-2-carboxylic acid; DHI, 5,6-dihydroxy indole; IQCA, indole-5,6-quinone carboxylic acid; IQ, indole-5,6-quinone. Adapted from [9].

Skin pigmentation can develop as a result of intrinsic and extrinsic pathways, including exposure to ultraviolet (UV) radiation, pollution, drugs, or chemicals [5,6]. Melanin, a pigment that contributes to the pigmentation of skin, hair, and eyes as well as photoprotection, is overproduced in hyperpigmented skin [5,6,7]. Skin hyperpigmentation can have different clinical manifestations, such as freckles, solar lentigo, and melasma, which can pose aesthetic issues [6,8]. Depigmenting agents are widely used for the treatment of hyperpigmentation in order to lessen or limit pigmentation by acting on several melanogenesis processes, like tyrosinase inhibition [5,7]. Tyrosinase is a key enzyme in the process of synthesizing melanin, the pigment responsible for skin pigmentation, as shown in Figure 1.

Many ingredients act on tyrosinase activity, transcription, or gene expression. Kojic acid is commonly used as the standard depigmenting ingredient with high skin whitening potency and is widely used in cosmetic and pharmaceutical formulations [10]. However, this compound has some limitations regarding possible side effects, namely contact dermatitis, allergies, or increased skin sensitivity [10]. Therefore, it is important to find alternatives for skin depigmenting agents. Recently, new depigmenting ingredients have emerged, such as bakuchiol, tranexamic acid, and 4-butylresorcinol, and others have increased their use over the years, including ascorbic acid and its derivatives [11]. α-Arbutin has also been widely used instead of hydroquinone, a well-known depigmenting agent that is no longer permitted in cosmetic products due to safety issues, including the fact that its use causes irritant contact dermatitis and ochronosis [12].

α-Arbutin is a polyphenol produced by microorganisms or microbial glycosyltransferases by glycosylation of hydroquinone that either directly suppresses the activity of melanosomal tyrosinase or engages in substrate-mediated competition with tyrosinase for the active site [13,14]. Ascorbic acid reduces the enzymatically generated *o*-quinones, blocking the remaining oxidative chemical reactions in the process of melanogenesis [15,16,17,18]. Furthermore, ascorbic acid and its derivatives, namely ascorbyl glucoside, can interact with copper ions in the tyrosinase active site, explaining the tyrosinase inhibitor effect in vitro [19,20]. Bakuchiol, a functional retinol analogue, is a free radical scavenger with a similar mechanism of skin depigmentation, modulating several skin retinoid-responsive genes [11,21,22]. The depigmenting activity of bakuchiol has also been linked to the reduction of pre-formed melanin and the suppression of α-melanocyte-stimulating hormone activation [23]. Tranexamic acid interferes with the interplay between melanocytes and keratinocytes by inhibiting the plasminogen/plasmin pathway [11,24,25,26]. This results in a reduction in free arachidonic acid and the formation of prostaglandins, which are inflammatory mediators and melanocyte stimulators [24,25,26]. Moreover, tranexamic acid exhibits structural similarities to tyrosinase, leading to the hypothesis that this compound may act as a competitive antagonist for the enzyme, thereby enhancing its depigmenting effect [26]. Finally, 4-butylresorcinol is a potent tyrosinase and tyrosinase-related protein-1 (TRP-1) inhibitor, reducing melanin production [7,11,27].

Nevertheless, no comparative studies have been conducted to assess both the tyrosinase inhibitory activity and the concurrent safety of various depigmenting agents, including bakuchiol, a novel natural skin-lightening agent. These studies can provide important knowledge on the most safe and potent tyrosinase inhibitors, helping formulators develop effective cosmetic products and assisting chemists in developing innovative agents.

Phototoxicity is an acute light-induced skin irritation when photoreactive chemicals are topically or systemically applied. The main event in any phototoxic reaction is the absorption of photons of a wavelength that induces the excitation of the chromophore, and the excitation energy is often transferred to oxygen molecules, followed by the generation of reactive oxygen species (ROS) [28]. Oxidative stress caused by high amounts of ROS can impair cellular macromolecules, such as DNA, lipids, and proteins, leading to cytotoxicity [28]. A ROS assay protocol was proposed and developed to screen the photoreactivity of chemicals through the generation of the superoxide anion (by an electron transfer reaction: type I reaction) and singlet oxygen (by energy transfer: type II reaction), and their generation is an early-stage chemical reaction as part of the phototoxicity mechanism [29]. As the scientific literature lacks information on the photoreactivity of most depigmenting agents focused on in this study, despite these compounds being included in cosmetic formulations applied to skin exposed to solar radiation, it is crucial to investigate this parameter to assess their photostability.

Skin safety is also a very critical factor when it comes to cosmetic ingredients. Regarding depigmenting ingredients that can act directly on the various stages of melanogenesis, namely tyrosinase inhibition, it is important to assess the toxicity of these compounds on melanocytes. Thus, in this work, we aim to compare depigmenting active ingredients that have been more recently incorporated into cosmetic formulations. We will assess their in vitro tyrosinase inhibitory activity, in vitro cytotoxicity for melanocytes, and in vitro photoreactivity. Our goal is to identify the depigmenting agent that exhibits the highest activity against tyrosinase, the lowest toxicity to melanocytes, and the least photoreactivity, thus presenting the most promising candidate for depigmentation in cosmetic applications.

## 2. Results and Discussion

Cosmetic products can incorporate depigmenting active ingredients to reduce skin melanin, which is the pigment responsible for skin pigmentation [30]. These ingredients can function through several mechanisms, although the most common is tyrosinase inhibition, a crucial enzyme in the melanogenic pathway [30]. Depigmenting agents have witnessed significant demand and are often employed in combination in cosmetic products. Therefore, it is crucial to ascertain which of these agents is not only the safest but also the most effective in achieving their intended purposes. Therefore, in a first approach, we compared various depigmenting agents used in cosmetic products, namely α-arbutin (ABT), ascorbic acid (AA), ascorbyl glucoside (AA-2G), bakuchiol (BAK), 4-butylresorcinol (4BR), and tranexamic acid (TA), regarding their photoreactivity and tyrosinase inhibitory activity. Ultimately, the safety profile of the compounds was evaluated in melanocytes.

### 2.1. UV-Visible Spectra 

**Figure 2:**
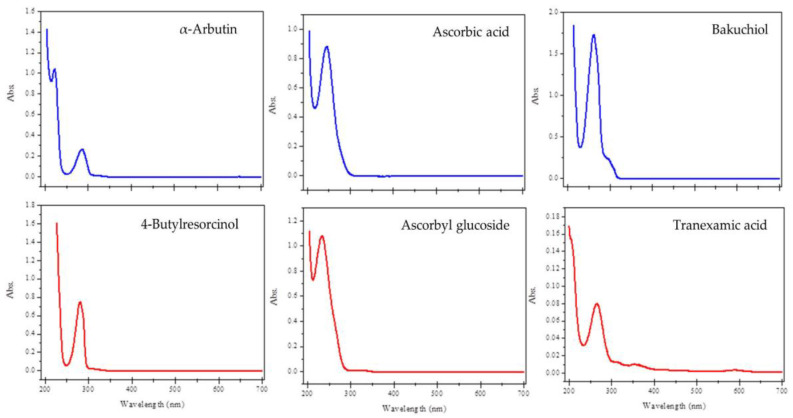
UV-visible spectra of the different depigmenting agents.

The phototoxic potential of chemicals is related to the photochemical properties of compounds, especially light absorption within the range 290–700 nm and the generation of reactive species following absorption of UV-visible light [29]. If a compound does not have a molar extinction coefficient (MEC) or molar absorptivity greater than 1000 L⋅mol^−1^ cm^−1^ at any wavelength between 290 and 700 nm, it is not considered to be sufficiently photoreactive to result in direct phototoxicity [29]. Thus, the UV-visible spectra of the depigmenting agents were determined, and the results showed that all compounds absorb radiation within the spectral range of 200 to 700 nm (Figure 2). The studied depigmenting agents contain in their molecular structures π systems (some of them conjugated), phenolic and/or hydroxyl, carbonyl, and amine groups. The absorption peaks observed in the UV-visible spectra can be assigned to π-π* and n-π* electronic transitions.

As the wavelength of maximum absorbance obtained for all compounds was below 290 nm, the MEC value for each depigmenting compound was calculated at this wavelength (Table 1).

The results show that all the tested depigmenting agents had a MEC value greater than 1000 L mol^−1^ cm^−1^, except for TA. Consequently, additional photosafety testing is not recommended for TA, as there is no anticipated photoreactivity and the likelihood of phototoxicity is minimal.

### 2.2. ROS Assay

In addition to light absorption, the generation of reactive species from chemicals following the absorption of UV-visible light is seen as a key determinant for causing direct phototoxic reactions [31]. The ROS assay can be used as an in chemico screening tool for evaluating the photoreactivity of chemicals. Therefore, the ROS assay was performed to evaluate the photoreactivity of the depigmenting agents ABT, AA, AA-2G, BAK, and 4BR (Table 2). These depigmenting agents were tested at 200 μM (final concentration) to identify precipitation, coloration, or any other interference in the reaction mixture. All substances tested were soluble in dimethyl sulfoxide (DMSO) or 20 mM sodium phosphate buffer (NaPB, pH 7.4) except for BAK, which showed precipitation in the reaction mixture for singlet oxygen (SO) and superoxide anion (SA) even at the lowest concentration of 20 μM.

AA-2G revealed low ROS generation when exposed to radiation, below the threshold levels for singlet oxygen (1 ± 1) and for superoxide (1 ± 0), and therefore could be classified as non-photoreactive. ABT showed high levels of singlet oxygen (19 ± 1), being close to the cut-off value (^1^O_2_ ≥ 25; O_2_^•−^ ≥ 20). Although ABT cannot be classified as photoreactive, it is necessary to be aware of its behavior when exposed to more intense radiation and for extended periods of time. The same principle could be applied to 4BR, which presented high levels of superoxide (13 ± 1), close to the threshold level. For AA, the values obtained for singlet oxygen and superoxide were quite high. However, this compound is known to interfere with the reactions involved in the ROS assay, as it reduces the tetrazolium salt to a formazan directly while accelerating the oxidation of imidazole derivatives, causing a false positive result [31,32]. The difference between the results obtained for AA (inconclusive/false positive) and AA-2G (non-photoreactive) can be explained by the fact that AA-2G is a stable derivative of AA, resisting oxidation and reduction reactions [33]. In the case of BAK, it was not possible to conduct the ROS assay due to solubility issues; however, it is necessary to assess its phototoxicity since it has a high MEC value (5648 L mol^−1^ cm^−1^).

### 2.3. Inhibition of Tyrosinase Activity

For the tyrosinase inhibition assay, an initial screening at 150 µM for each compound was assessed, and serial dilutions were carried out in the cases where activity was high enough to allow IC_50_ determination. The results are presented as a percentage of tyrosinase inhibition at a concentration of 150 µM and, when possible, as an IC_50_ value, and kojic acid was used as a positive control (Table 3). 4BR was found to be a significantly more potent inhibitor compared to the standard kojic acid, which is commonly used in cosmetic formulations as a skin whitening agent. Additionally, BAK showed 52.59% inhibition, with a standard deviation of 2.04, too close to 50% at the maximum concentration assayed to allow a correct determination of IC_50_. The other compounds tested presented a lower inhibitory effect, with 11.17, 13.20, and 27.04% tyrosinase inhibition at 150 µM for AA-2G, TA, and ABT, respectively.

The results obtained for ABT and BAK were similar to the data found in the literature concerning percentage of inhibition (ABT ≈ 30% at 125 µM) and IC_50_ values (BAK = 37.22 ± 5.18 µM at 1 mM of L-tyrosine) [23,34,35]. The IC_50_ value obtained for 4BR is lower than those found in the literature (21 μM and 13.5 μM) [27]. The low tyrosinase inhibition by AA-2G and TA are consistent with the data obtained in the literature, where inhibition only occurs at relatively high concentrations [25,36,37,38]. AA at 150 µM prevented the formation of the chromophore by 58.70%. However, this result cannot be interpreted uniquely as its ability to inhibit tyrosinase, since AA may also reduce the enzymatically generated *o*-quinones [16].

**Figure 3:**
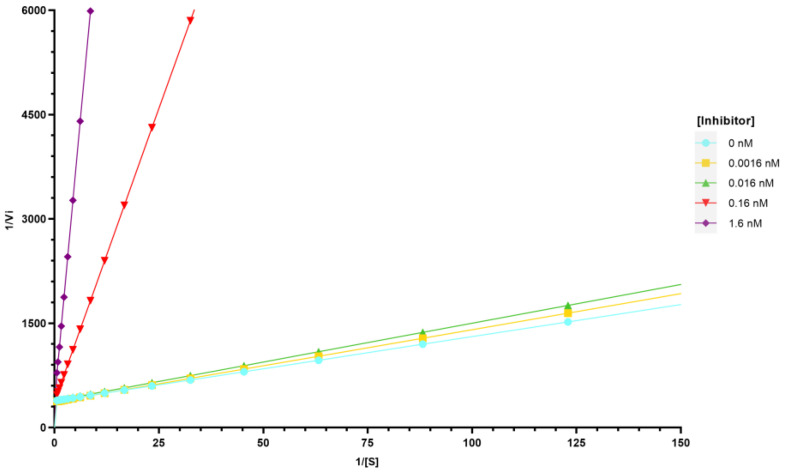
Lineweaver-Burk plot for 4BR inhibition of tyrosinase.

Further experiments were carried out to determine the type of tyrosinase inhibition evoked by 4BR, since it was the most potent inhibitor of all compounds. Tyrosinase inhibitors can be classified as competitive, uncompetitive, mixed-type (competitive/uncompetitive), and non-competitive [39]. Competitive inhibitors bind to the active site of the enzyme, preventing the substrate from binding, and can be reversed by adding more substrate [39]. An uncompetitive inhibitor can only bind to the enzyme-substrate complex, while the mixed-type can bind to both the active site and the enzyme-substrate complex [39]. Non-competitive inhibitors can bind to the enzyme in a region different from the active site or to the enzyme-substrate complex, preventing its activity [39]. The results presented in Figure 3 showed that 4BR is a competitive inhibitor, where the maximal velocity (V_max_) value is constant, while the Michaelis–Menten constant (K_m_) increases with the concentration of the inhibitor at a fixed substrate concentration.

**Figure 4:**
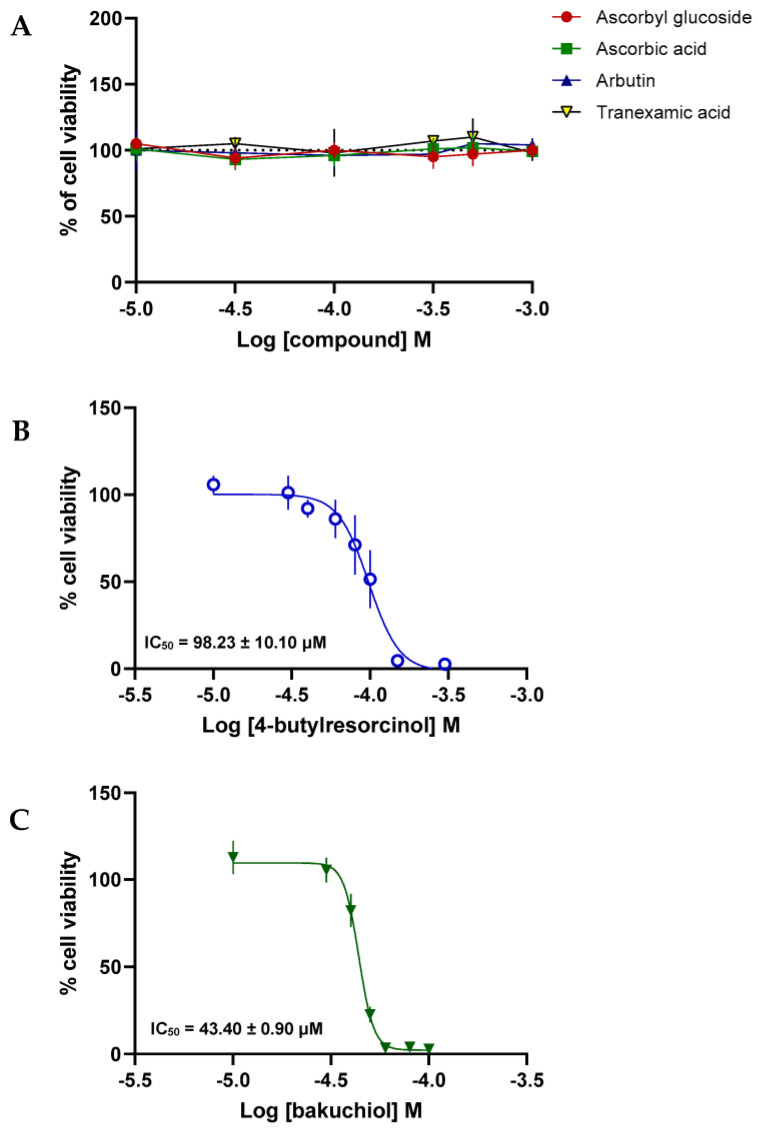
Concentration-response curves to evaluate the cytotoxicity of the depigmenting compounds (**A**–**C**) in B-16V melanocytes. Values are means ± SDM from at least three independent experiments.

In order to evaluate the skin safety profile of the depigmenting compounds, the viability of B-16V melanocytes was assessed after being treated with increasing concentrations of AA-2G, AA, ABT, TA, 4BR, and BAK. Considering the limit of solubility of the compounds in water and in DMSO, a high range of concentrations, including the IC_50_ of the tyrosinase inhibition of the different compounds previously determined, was selected (0–300 µM in DMSO for 4BR and BAK, and 10–1000 µM in water for the other compounds). While AA-2G, AA, ABT, and TA did not interfere with cell viability (Figure 4A), both 4BR (Figure 4B) and BAK (Figure 4C) reduced cell viability in a concentration-dependent manner, with an IC_50_ of 97.64 ± 11.56 μM and 43.40 ± 0.90 μM, respectively. Given that ABT is a glycosylated derivative of hydroquinone, there is a potential for the release of hydroquinone in vivo. The safety assessment of hydroquinone indicates that this depigmenting agent initiates growth inhibition of B16 mouse melanoma cells at a concentration of 0.625 µg/mL, achieving complete inhibition at concentrations ranging from 1.25 to 2.5 µg/mL [40]. In contrast, ABT did not interfere with cell viability, even at its highest concentration of 1000 µM, which notably exceeded the cytotoxic concentrations found for hydroquinone. The tested depigmenting agents have different potencies for tyrosinase activity inhibition, with distinct mechanisms of action responsible for this activity. Thus, it is relevant to associate non-cytotoxic concentrations of depigmenting ingredients with different mechanisms of action that have a synergistic effect in formulations to improve the outcomes of the treatment of skin hyperpigmentation [41].

## 3. Materials and Methods

### 3.1. Depigmenting Agents

The depigmenting agents were selected according to their reported application in cosmetic products and ability to inhibit tyrosinase. 4BR and BAK were gifts from DKSH Portugal Unipessoal Lda. (Maia, Portugal). TA was obtained from Fagron Ibérica (Barcelona, Spain). AA-2G and ABT were offered by Ascend Biotech LLC (Florham Park, NJ, USA). AA was obtained from Sigma-Aldrich (St. Louis, MO, USA).

### 3.2. Chemicals

Sodium phosphate monobasic monohydrate was obtained from Fluka (Buchs, Switzerland). Sodium phosphate dibasic anhydrous and methanol were obtained from Carlo Erba Reagents (Val-de-Reuil, France). Imidazole, *p*-nitroso dimethylaniline (RNO), and nitro blue tetrazolium chloride (NBT) were purchased from Alfa Aesar (Ward Hill, MA, USA). Tyrosinase from *Agaricus bisporus*, L-tyrosine, kojic acid, and DMSO were obtained from Sigma-Aldrich (St. Louis, MO, USA).

### 3.3. UV-Visible Spectral Analysis

UV-visible spectral analysis was conducted as previously described [28]. The depigmenting agents were dissolved in methanol or 20 mM sodium phosphate buffer (NaPB, pH 7.4) at 10 μg/mL (final concentration). The UV-visible absorption spectra were analyzed using a Shimadzu UV-1700 spectrophotometer (Shimadzu Corp., Kyoto, Japan) and UV-transparent quartz cuvettes (Hellma, Müllheim, Germany) with a 10 mm path length. Each spectrum was corrected for solvent-specific baseline absorption. Molar extinction coefficients (MEC) were calculated using the highest absorption peaks between 290 and 700 nm at 10 μM.

### 3.4. ROS Assay

The ROS assay was performed as previously described in Aguiar et al. [28] and the OECD ROS assay for photoreactivity protocol [29]. Stock solutions of all substances were prepared at 10 mM in DMSO or 20 mM sodium phosphate buffer (NaPB, pH 7.4) and used within the same day. SO generation was detected by spectrophotometric measurement of RNO bleaching, followed by a decrease in the absorbance of RNO at 440 nm. SA generation was detected by observing the reduction of NBT to NBT^+^, the formation of which can be monitored spectrophotometrically at 560 nm. The depigmenting agents were tested at a final concentration of 200 μM. When precipitation was observable (using a microscope) before light exposure, a lower concentration (20 μM) of the depigmenting agents was tested.

Photoreactivity of the test substances was evaluated according to the result (mean of triplicate determinations) from the ROS assay [29]: a test substance was classified as a photoreactive substance when an SO value of 25 or more and/or an SA value of 20 or more was measured; in turn, it was considered to be a non-photoreactive substance when values of less than 25 for SO and less than 20 for SA were recorded.

### 3.5. Tyrosinase Inhibition Assay

Tyrosinase inhibition assays were carried out as described in Rosa et al. [42]. Briefly, 25 μL of tyrosinase enzyme solution (135 U/mL), 25 μL of ten serial concentrations of the compounds (dissolved in 0.1 M phosphate buffer pH 6.8 containing no more than 2.5% DMSO, yielding final concentrations of 0.3–150 μM), and 100 μL phosphate buffer were mixed in a 96-well plate and incubated at 37 ± 2 °C for 20 min. Then, 50 μL of 1.66 mM of tyrosine solution in 0.1 M phosphate buffer (pH 6.8) were added. Enzyme activity was measured at 490 nm every 10 min for 30 min in a Bio-Rad Model 680 Microplate Reader (Bio-Rad Laboratories, Inc., Hercules, CA, USA). In cases where the samples were very active at the minimum concentration tested, lower concentration ranges were assayed. Kojic acid was used as a positive control. The experiments were conducted in triplicate. For each concentration, enzyme activity was calculated as a percentage of the velocities compared to those of the assay using buffer without any inhibitor. The IC_50_ value was determined as the concentration of the compound that inhibited 50% of enzyme activity. The type of inhibition and inhibition parameters of the compounds, i.e., the K_m_ and V_max_, were determined by Lineweaver–Burk plot analysis using various concentrations of L-tyrosine.

### 3.6. Cell Viability

B-16V cells (DSMZ ACC-370, Braunschweig, Germany) were cultured in Dulbecco’s Modified Eagle’s Medium (DMEM) containing 3.7 g/L NaHCO_3_, 1.0 g/L d-glucose, 1 mM sodium pyruvate, and stable glutamine, supplemented with 10% (*v*/*v*) heat-inactivated fetal bovine serum (FBS), 50 U/mL penicillin, and 50 μg/mL streptomycin (all reagents from PAN-Biotech GmbH, Aidenbach, Germany). The cells were cultivated at 37 °C in a humidified atmosphere of 95% air and 5% CO_2_. Subculturing was performed twice a week, when the cells reached 70–80% confluence, through cell trypsinization with 0.25% Trypsin and 1 mM EDTA.4Na (PAN-Biotech GmbH, Aidenbach, Germany).

To evaluate cell viability, B-16V cells were seeded at a cell density of 3 × 10^4^ cells/mL in 96-well plates and allowed to proliferate for 48 h. The cells were treated with the depigmenting compounds, AA-2G, AA, ABT, TA, 4BR, and BAK, at increasing concentrations, or with the respective solvent, H_2_O, or DMSO (maintained below 0.15% (*v*/*v*) in all conditions tested), for 48 h. Then, PrestoBlue^®^ reagent (Invitrogen, Waltham, MA, USA) was added to each well. When entering a living cell, the PrestoBlue^®^ reagent is reduced from resazurin to resorufin, whose fluorescence is proportional to the number of metabolically active cells. Fluorescence was measured at an excitation/emission wavelength of 530/590 nm in an automated microplate reader (Synergy HT, Biotek Instruments Inc., Winooski, VT, USA).

All conditions were performed in triplicate, and the results of at least three independent experiments were expressed as a percentage of the control (solvent). GraphPad Prism 9.5.1 was used to fit non-linear regression to the data and calculate the IC_50_ values of depigmenting compounds for B-16V cell viability.

## 4. Conclusions

A notable gap exists in research regarding the comparative analysis of various depigmenting compounds, which could hold substantial value for cosmetic product formulators. Furthermore, certain depigmenting agents are being used without the support of sufficient scientific evidence regarding their effectiveness and stability. Additionally, some of these agents may present limitations, including skin adverse effects. These challenges make the process of selecting the most suitable depigmenting ingredients for formulations designed to lighten the skin difficult. In order to fill this gap, in this work, a ROS assay was employed to evaluate the photoreactivity and explore the photostability of different skin depigmenting agents when exposed to simulated sunlight. According to the results of the ROS assay, the depigmenting agents are thought to be photostable. However, ABT and 4BR showed more sensitivity to radiation, producing higher levels of ROS than the other compounds. The different depigmenting compounds tested can then be ranked from the least photoreactive/photostable to the most photoreactive/photostable: TA < AA-2G < 4BR < ABT < AA. The ROS assay allowed for the determination of the photostability of depigmenting agents, although further studies are needed to assess the photosafety of these ingredients in light-exposed skin, namely phototoxicity, photoallergy, photogenotoxicity, and photocarcinogenicity tests.

The same compounds were also subjected to an in vitro test to assess their tyrosinase inhibitory activity, which showed that 4BR was the most potent tyrosinase inhibitor among the tested ingredients. Based on the results obtained for the percentage of tyrosinase inhibition at 150 µM, the compounds can be placed in order from highest to lowest inhibition produced: 4BR > BAK > ABT > TA > AA-2G. Despite being recognized as a tyrosinase inhibitor, in this study, the potency of AA was inconclusive. In the mushroom tyrosinase assay, AA can chemically prevent the progression of melanogenic reactions that lead to the formation of the quantifiable chromophore. Therefore, the effect cannot be uniquely interpreted as a tyrosinase inhibitory effect. This chemical interference highlights some limitations of the mushroom tyrosinase assay that could be overcome by using the human enzyme.

Tyrosinase is a key enzyme in melanin production. Therefore, identifying compounds that effectively inhibit melanin synthesis from the beginning of the biochemical process may contribute to increasing the effectiveness of depigmenting formulations.

BAK and 4BR reduced the viability of B-16V melanocytes in a concentration-dependent manner, unlike the other depigmenting agents. BAK may present a critical margin of safety for this particular mechanism of action (tyrosinase inhibition) since the IC_50_ value for melanocyte viability was far below the concentration tested in the in vitro tyrosinase inhibition assay. However, this compound can have depigmenting activity acting by other mechanisms such as free radical scavenging and by increasing cell turnover, as described in the scientific literature, in a concentration between 0.5 and 1% in cosmetic products, which is within the margin of safety for melanocytes [43]. Given the results of melanocyte viability in the presence of the different depigmenting ingredients, these can be ordered from the safest to the least safe for those cells: AA-2G, AA, ABT, TA > 4BR > BAK.

Overall, 4BR was the most effective compound inhibiting tyrosinase, showing adequate photostability despite being one of the depigmenting compounds with the highest toxicity to melanocytes. However, no cytotoxic effect was observed in the concentration range encompassing the IC_50_ of 4BR for tyrosinase activity.

The insights obtained in this study offer valuable scientific guidance concerning the photostability, tyrosinase inhibitory efficacy, and safety of depigmenting agents. This information can greatly inform the development of cosmetic products aimed at addressing hyperpigmentation in the cosmetics industry. This study allowed us to understand which are the most potent and photostable tyrosinase inhibitors, even considering differences between human and mushroom tyrosinases [44], as well as the concentrations at which they can be used to ensure skin safety. Considering the existence of advances in the obtention of human tyrosinase by heterologous expression in sufficient amounts, which will solve the difficulties of using the human enzyme as a model, it is recommended that in the future most studies concerning skin depigmenting agents should be carried out using human tyrosinase.

Nevertheless, because depigmenting compounds may act through other mechanisms of action rather than tyrosinase inhibition, future studies should also explore their relative efficacy at different depigmenting mechanisms. This will expand the breadth of knowledge needed to define the optimal combination of depigmenting compounds in a final formulation.

This knowledge will also serve as a foundation for health professionals’ recommendations and is also instrumental in recognizing innovation opportunities within the cosmetic and chemical industries.

## Figures and Tables

**Table 1:** Molar extinction coefficient (MEC) at 290 nm of the tested compounds.

Compound	Absorbance (10 µg mL^−1^)	MEC (L mol^−1^ cm^−1^)
ABT	0.0736	ε = 2004
AA	0.1586	ε = 2793
AA-2G	0.0303	ε = 1025
BAK	0.2203	ε = 5648
4BR	0.2088	ε = 3471
TA	0.0242	ε = 380

**Table 2:** ROS assay for singlet oxygen (^1^O_2_) and superoxide anion (O_2_^•−^) (mean ± SEM, n = 2).

Compound	^1^O_2_	O_2_^•−^
ABT	19 ± 1	0 ± 0
AA	671	26
AA-2G	1 ± 1	1 ± 0
BAK	inconclusive	inconclusive
4BR	1 ± 0	13 ± 1

**Table 3:** Inhibition of tyrosinase activity is expressed as a percentage of inhibition (n = 3).

Compound	% Inhib (150 µM)	SD	IC_50_ (µM)	SD
TA	13.20	1.99	-	-
BAK	52.59	2.04	-	-
AA-2G	11.17	0.84	-	-
ABT	27.04	1.74	-	-
4BR *	100	n.d.	5.8 × 10^−5^	0.8 × 10^−5^
Kojic acid **	99.15	0.64	12.80	0.01

* 4BR: IC_50_ = 5.8 × 10^−5^ ± 0.8 × 10^−5^ µM; ** standard inhibitor compound.

## Data Availability

Data are contained within the article.
